# Assessing the origin, genetic structure and demographic history of the common pheasant (*Phasianus colchicus*) in the introduced European range

**DOI:** 10.1038/s41598-021-00567-1

**Published:** 2021-11-05

**Authors:** Mohammad Reza Ashrafzadeh, Rasoul Khosravi, Carlos Fernandes, Cecilia Aguayo, Zoltán Bagi, Vukan M. Lavadinović, László Szendrei, Dejan Beuković, Bendegúz Mihalik, Szilvia Kusza

**Affiliations:** 1 Department of Fisheries and Environmental Sciences, Faculty of Natural Resources and Earth Sciences, Shahrekord University, 8818634141 Shahrekord, Iran; 2 Department of Natural Resources and Environmental Engineering, School of Agriculture, Shiraz University, 71441-13131 Shiraz, Iran; 3 cE3c - Centre for Ecology, Evolution and Environmental Changes, Departamento de Biologia Animal, Fac-Uldade de Ciências, Universidade de Lisboa, 1749-016 Lisboa, Portugal; 4 Faculdade de Psicologia, Universidade de Lisboa, Alameda da Universidade, 1649-013 Lisboa, Portugal; 5 Agrarian University of Ecuador, Guayaquil, 090104 Ecuador; 6 Centre for Agricultural Genomics and Biotechnology, University of Debrecen, Debrecen, 4032 Hungary; 7 Faculty of Forestry, University of Belgrade, 11030 Belgrade, Serbia; 8 Department of Nature Conservation Zoology and Game Management, University of Debrecen, Debrecen, 4032 Hungary; 9 Faculty of Agriculture, University of Novi Sad, 21000 Novi Sad, Serbia; 10 Institute of Genetics and Biotechnology, Gödöllő Campus, Hungarian University of Agriculture and Life Sciences, Gödöllő, 2100 Hungary

**Keywords:** Ecology, Genetics

## Abstract

The common pheasant, a game species widely introduced throughout the world, can be considered as an ideal model to study the effects of introduction events on local adaptations, biogeographic patterns, and genetic divergence processes. We aimed to assess the origin, spatial patterns of genetic variation, and demographic history of the introduced populations in the contact zone of Central and Southeast Europe, using mitochondrial DNA control region sequences and microsatellite loci. Both types of molecular markers indicated relatively low to moderate levels of genetic variation. The mtDNA analyses revealed that common pheasants across the study area are divided into two distinct clades: B (*mongolicus* group) and F (*colchicus* group). Analyses of the microsatellite data consistently suggested a differentiation between Hungary and Serbia, with the pheasant population in Hungary being much more genetically homogeneous, while that of Serbia has much more genetic mixture and admixture. This cryptic differentiation was not detected using a non-spatial Bayesian clustering model. The analyses also provided strong evidence for a recent population expansion. This fundamental information is essential for adequate and effective conservation management of populations of a game species of great economic and ecological importance in the studied geographical region.

## Introduction

Genetic variation plays a central role in the evolution and ability of species to adapt to continuous environmental changes, and in their long-term persistence in the face of an increasing and constant anthropogenic disturbances^1,2^. Characterizing the genetic diversity of populations, understanding the spatial patterns of this variation in the landscape, and investigating possible historical and current causes for these patterns, is fundamental knowledge for an informed conservation management of wildlife, which is especially critical for threatened populations^3–5^.

The spatial patterns of genetic variation and population structure of species may be influenced by geographic distance^6^, natural and anthropogenic barriers^7–9^, migration behaviour and sex-biased dispersal^10^, climate change^11^, and human introductions and hybridization^12–14^. The introduction of species to areas outside of their historic range is a widespread management action to support harvest in wildlife, fisheries, and forestry^15^. However, translocating species may lead to genetic admixture between taxa due to hybridization, and therefore change spatial patterns of genetic variation and population structure of native species, or cause gradual dilution of autochthonous genetic diversity and reduce individual fitness due to outbreeding^1,16^. Unrecorded releases of individuals from different subspecies for hunting purposes , besides possibly being detrimental to natural populations (e.g. outbreeding depression), further complicate our understanding of the genetic profile of introduced populations^17–20^.

The common pheasant (*Phasianus colchicus* L.), a non-migratory gallinaceous bird, can be considered as an ideal model for studying the effects of introduction events on local adaptations, biogeographic patterns, and possible speciation processes^21^. The common pheasant, with 31 described subspecies based on morphology, colour patterns, and range discontinuities^22^, is considered native from eastern Siberia to China, Iran, the Caucasus and the south-eastern Balkans^22,23^. The subspecies inhabit significantly different landscapes and climatic niches, from montane ecosystems to isolated oases in semi-desert regions, exhibiting distinct phenotypes and genotypes^24,25^. Although the common pheasant is widespread and relatively common over most of its range, local and regional populations are facing declines mainly due to over-hunting and habitat loss^26^. For example, *P. c. talischensis*, from Azerbaijan and northwest Iran bordering the southwest of the Caspian Sea, is now a very rare subspecies^27^ comprising a total population of 200–300 individuals^28^.

The common pheasant has been introduced to Europe, North America, and Australia^26^. The first known introductions of the species into Europe, consisting of South Caucasus pheasants (*P. c. colchicus*), date back to 500–800 AD^29^. Much later, already in the eighteenth century, Mongolian (*P. c. mongolicus*) and Chinese ring-necked pheasants (*P. c. torquatus*) were also brought in to Europe^29^. The European populations are therefore descended from several subspecies and possibly from hybridization between them^22^. Therefore, the identity of these subspecies and the genetic composition of the introductions still need to be investigated because some common pheasant morphological subspecies are not supported by molecular data^30^, possibly as a result of recent range expansions and subspecies admixture^31^. Hybrids of different subspecies may also have been released in areas with native subspecies in Southeast Europe, which may lead to the extinction of the latter by genetic swamping^32^, as apparently in the case of the indigenous South Caucasus pheasant populations in Bulgaria^22^.

In Europe, the common pheasant is historically considered as one of the most important game species for recreational hunting^33^. It is especially prized by hunters for its meat and for being suitable for group hunting with dogs^34^. Non-random removal of individuals from wildlife populations by recreational hunting may affect the genetic structure, morphology, life history, and, ultimately, the viability of the target population^35–38^. Moreover, the common pheasant in Europe has long had extensive re-stocking to maintain populations and for hunting purposes, in which released individuals are mostly of non-local origin and farm-reared^17,22,24,33,39^. These individuals may have low genetic diversity, maladaptive alleles, and lower fitness in the local natural environment than indigenous individuals. Hybridization and introgression of maladaptive alleles into local populations can degrade autochthonous genetic heritage and disrupt coadapted gene complexes, making these populations less adapted to their environment^15^. It is likely that a large percentage of captive-bred individuals released recently and today, due to their possibly lower ecological fitness in foreign natural conditions, have been quickly removed by hunters and predators and generally have low survival and reproduction. Therefore, it is probable that the reproductive contribution of captive-bred individuals to the next generations is less than could be expected, and their greatest impact may be the transmission of livestock diseases and parasites. The combined effects of the selective removal of individuals (e.g. hunter bias for males) and hybridization and introgression with individuals of lower fitness are likely to have a profound impact on the evolution, genetic health, and long-term persistence of native game populations^16,37,38,40^.

It has been suggested that processes of introduction, stocking, and restocking in the common pheasant may lead to genetic differentiation between locations, likely enhanced by founder effects and genetic drift associated with small propagules, and that is subsequently maintained by limited interpatch gene flow^41^. Thus, the genetic structure of introduced common pheasant populations should be strongly shaped by founder effects, increased genetic drift, small to modest effective population sizes (Ne), and inbreeding^41^.

Despite the long history of the common pheasant as one of the most important game birds in Europe, nothing is known about the genetic variation and structure of the European populations. From a genetic point of view, the species has been studied essentially in terms of phylogeography and subspecies taxonomy throughout its distribution across Asia^30,31,42–44^, with the most recent data supporting eight major clades in this extant native range^45^. Evaluating genetic variation and structure in common pheasant populations, and identifying those with low and high levels of genetic diversity, is crucial for effective conservation and management of the species, for example to inform translocation efforts.

In this study, we begin the investigation of the origin, genetic diversity and structure, and demographic history of the common pheasant in Europe, with the analysis of populations in the contact zone between Central and Southeast Europe. We also compared the genetic diversity in the study area with that which has been reported in Asian populations, and assessed the hypothesis that the studied populations are derived exclusively from introductions from Asia. To this end, we analysed variation in a fragment of mitochondrial DNA (mtDNA), a molecular marker of excellence for phylogeography^46^ and for assessing the geographic origin of introduced lineages^47,48^, and in a set of polymorphic nuclear microsatellites, which are known to be powerful markers for studying genetic structure and processes at small temporal and spatial scales^49^. It is recognized that the combined analysis of mtDNA and microsatellites can be very informative for phylogeographic and population genetic studies of natural populations^50,51^.

## Results

### Mitochondrial DNA

#### Genetic diversity and phylogenetic analyses

**Figure 1:**
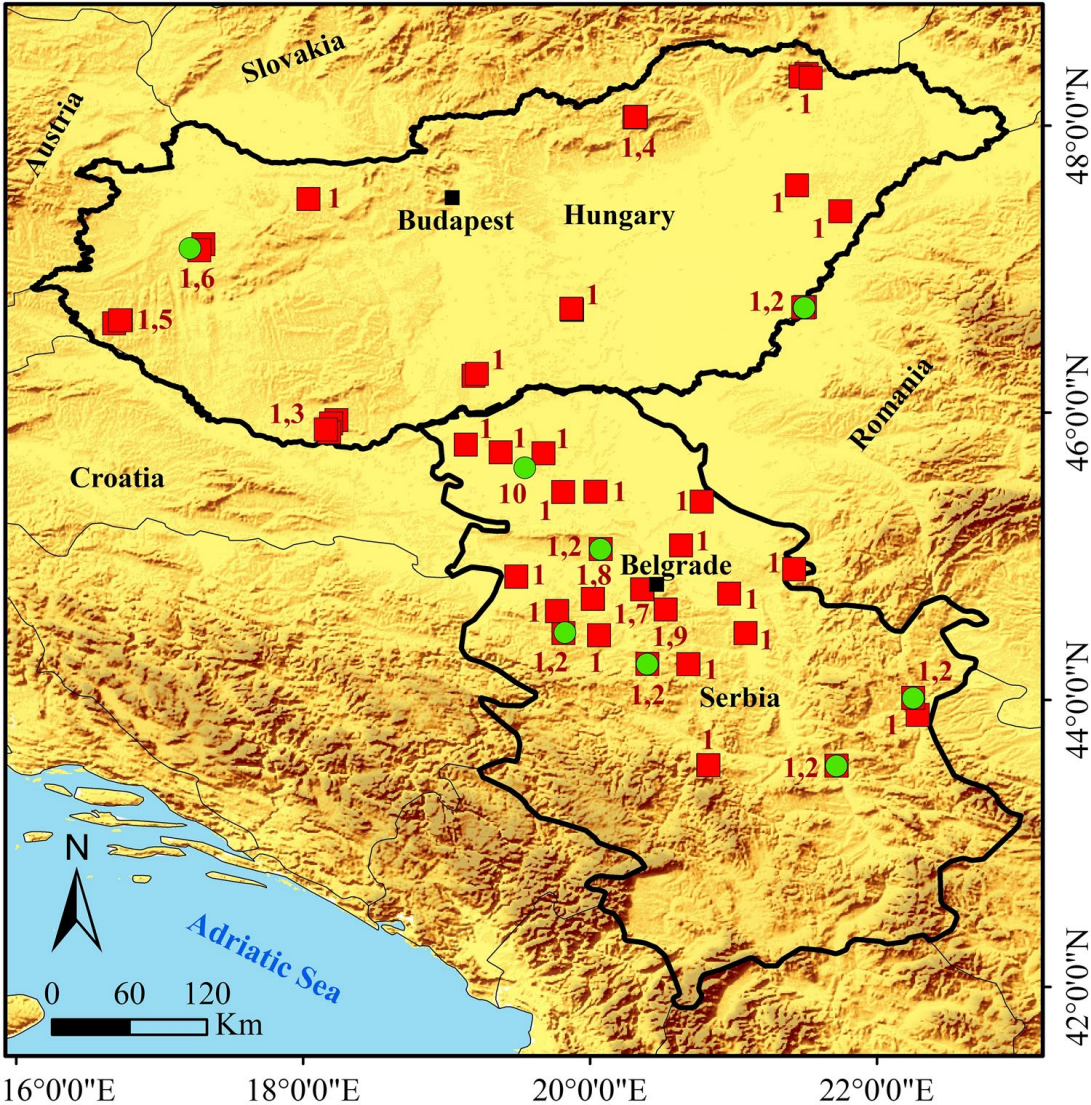
Sampling localities of the common pheasant in the contact zone of Central and Southeast Europe. Green circles and red squares indicate clades B and F, respectively, and the numbers next to these symbols denote the haplotypes found in each sampling locality, according to the numbering assigned to them (see Figs. 2 and 3 for details). Map source: ESRI. The map was generated using ArcGIS 10.4.1 by ESRI (available online at: http://www.esri.com/).

A 825 base pairs (bp) fragment of the mtDNA control region was sequenced for 69 *P. colchicus* individuals, 29 samples from Hungary and 40 samples from Serbia (Fig. 1). Additional sequences available on GenBank were included in the data set, resulting in a total of 271 sequences and 137 haplotypes (Table S1). Among our samples we detected 10 haplotypes, three previously reported and seven novel. In these 69 sequences, 20 of the 825 sites were polymorphic, nine of them singletons and 11 parsimony-informative.

**Figure 2:**
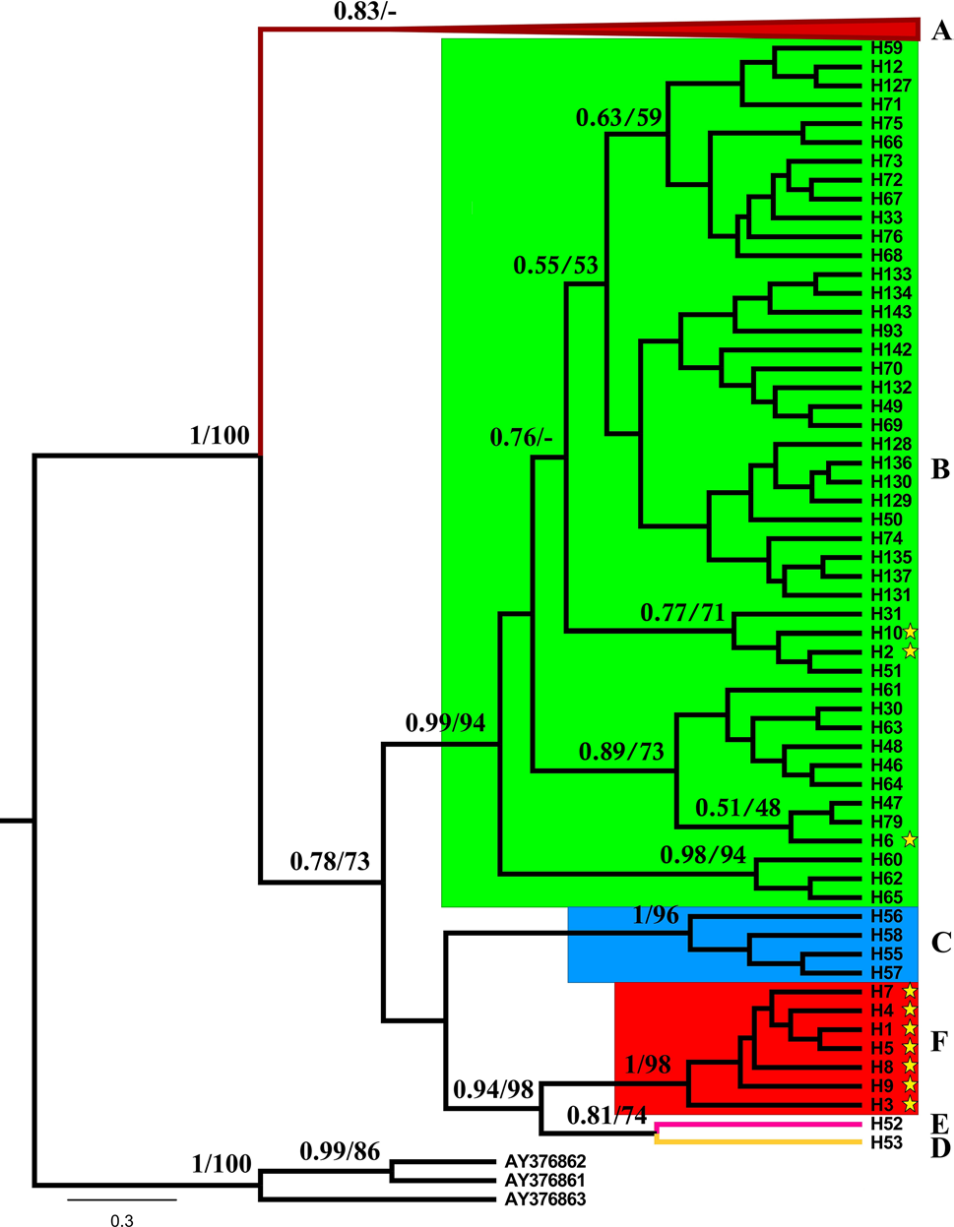
Phylogenetic relationships of common pheasant from the contact zone of Central and Southeast Europe with other common pheasants, based on the analyzed 825-bp fragment of the mtDNA control region and rooted with the green pheasant (*P. versicolor*; AY376861-3). The numbers on nodes are Bayesian posterior probabilities and ML bootstrap values, respectively. Yellow stars indicate haplotypes detected in this study. See Table S1 for details on haplotypes.

The ML and BI phylogenetic analyses of the 137 *P. colchicus* haplotypes plus the three *P. versicilor* outgroup sequences yielded identical topologies, indicating the presence of six main lineages with significant support from bootstrap values and Bayesian posterior probabilities (Fig. 2). Focusing on the 69 individuals analyzed here, the results suggest that the common pheasant mitochondrial haplotypes in the contact zone of Central and Southeast Europe belong exclusively to clades B and F (Fig. 2). Clade F is much more frequent and geographically widespread, while clade B is mainly present in Serbia, where the geographic overlap between the two clades is much more significant (Fig. 1). Within clade B, the new haplotypes H2 and H10, together with H31 (= haplotype C83 from Zhaosu, China, assigned to *P. c. mongolicus*;^42^) and H51 (previously detected by Qu et al.^42^ in an individual from Xianghai, China, assigned to *P. c. pallasi*, and in an individual from Libo, China, assigned to *P. c. decollatus*; see Table S1 for details) are closely related and form a small clade in the phylogenetic tree (Fig. 2). Haplotype H6, the third new haplotype in clade B, also formed a small clade with haplotypes H47 (= haplotype C66 from Anyang, China, assigned to *P.c. torquatus*;^42^) and H79 (= haplotype C24 from Xinyang, China, assigned to *P.c. torquatus*;^42^) (Fig. 2).

**Figure 3:**
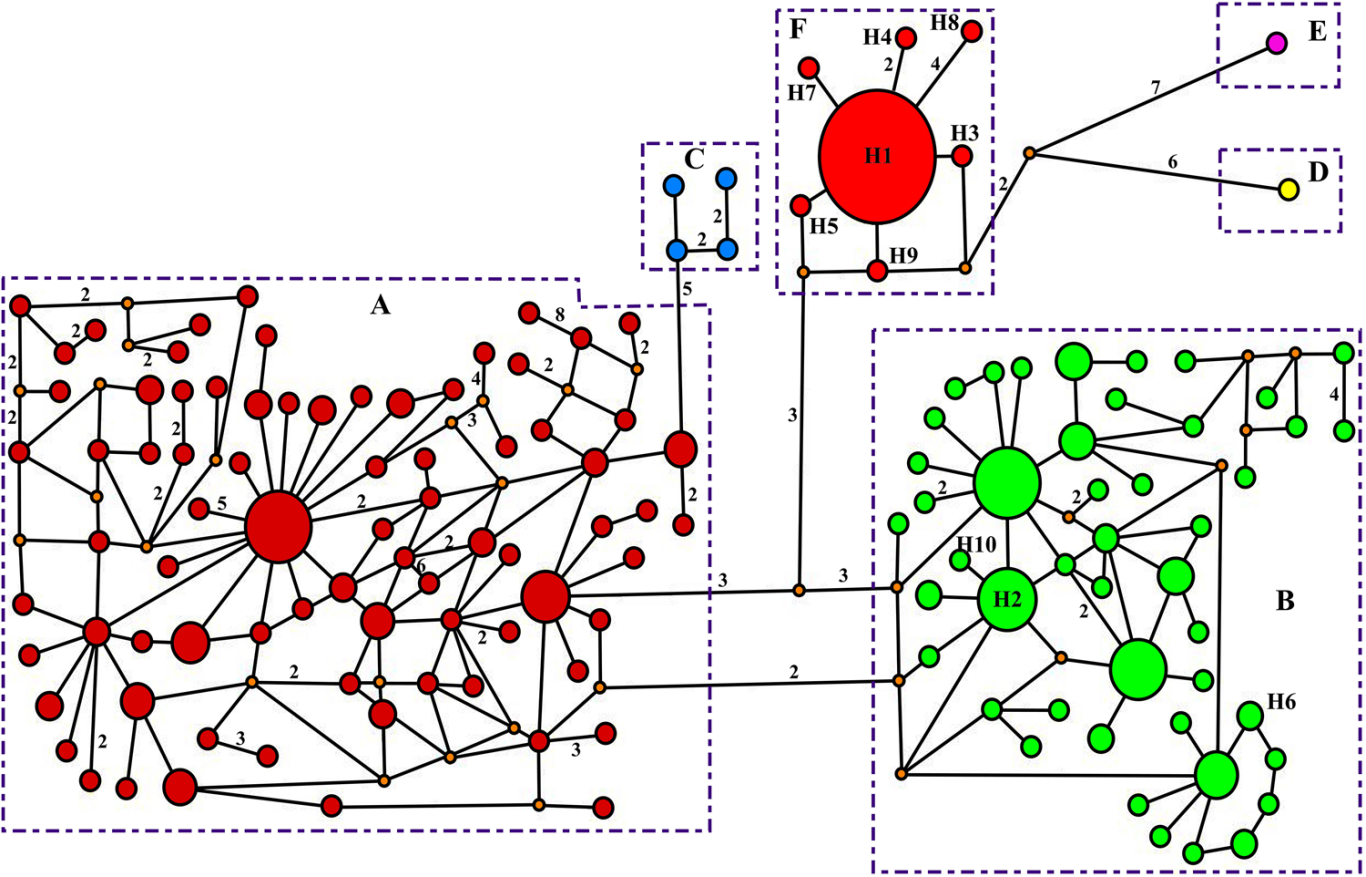
Median-joining haplotype network of common pheasant from the contact zone of Central and Southeast Europe and other common pheasants, based on the analyzed 825-bp fragment of the mtDNA control region. The sizes of the circles reflect the haplotype frequency, and the colours correspond to those of clades on Fig. 2. The groups of haplotypes corresponding to the clades in Fig. 2 are bounded by rectangles with dashed lines. Numbers next to the lines connecting haplotypes are present when the mutational steps separating them are > 1. Small orange circles represent undetected putative haplotypes. The 10 haplotypes found in this study are identified (H1-H10).

The median-joining network also recovered the haplotype groups identified in the phylogenetic trees (Fig. 3). The number of haplotypes in clades B (9 individuals) and F (60 individuals) were three and seven, respectively (Table 1). Haplotype H1 was the most common (Fig. 3) and being shared by 54 individuals across the study area. The second most common haplotype was H2, which was shared by seven individuals and also found over a relatively wide geographical area. The two main mitochondrial lineages exhibited relatively low haplotype and nucleotide diversities in the study area (Table 1).

**Table 1:** Genetic diversity summary statistics and results of neutrality tests and mismatch distribution analyses for clades (or BAPS clusters) B and F in the study area, based on the 825-bp fragment of the mtDNA control region.

	n	h	Hd (SD)	Π (SD)	K	P	Fu’s FS	Tajima's D	SSD	H_ri_
Clade B	9	3	0.417 (0.191)	0.00108 (0.00062)	0.8889	4	0.134	− 1.609*	0.025	0.255
Clade F	60	7	0.192 (0.068)	0.00041 (0.00019)	0.3333	10	− 5.411**	− 2.339**	0.001	0.482
Total	69	10	0.381 (0.073)	0.00322 (0.00076)	2.640	20	− 0.155	− 1.112	0.021	0.320

*n* number of samples, *h* number of haplotypes, *Hd* haplotype diversity, *π* nucleotide diversity, *K* mean number of nucleotide differences, *P* variable sites, *SD* standard deviation, *SSD* sum of squared differences in mismatch analysis, *H*_*ri*_ Harpending’s raggedness index.

*p < 0.05, significant; **p < 0.01, highly significant.

#### Population structure

The BAPS analysis assigned sequences of common pheasant from the study area to two clusters, where sequences belonging to each mitochondrial clade (B and F) fell into a distinct cluster (Figure S1). The AMOVA estimated that 96.07% of the mtDNA variation in the study area is due to the differentiation between the two populations (p < 0.0001) and, accordingly, the F_ST_ fixation index was extremely high (Table 2).

**Table 2:** AMOVA of the mtDNA variation in the study area taking into account the genetic structure represented by the two detected clusters (B and F).

Source of variation	d.f	Percentage of variation	Fixation index	p-value
Among populations	1	96.07	FST = 0.9607	p < 0.000
Within populations	67	3.94		
Total	68			

#### Demographic analyses and neutrality tests

**Figure 4:**
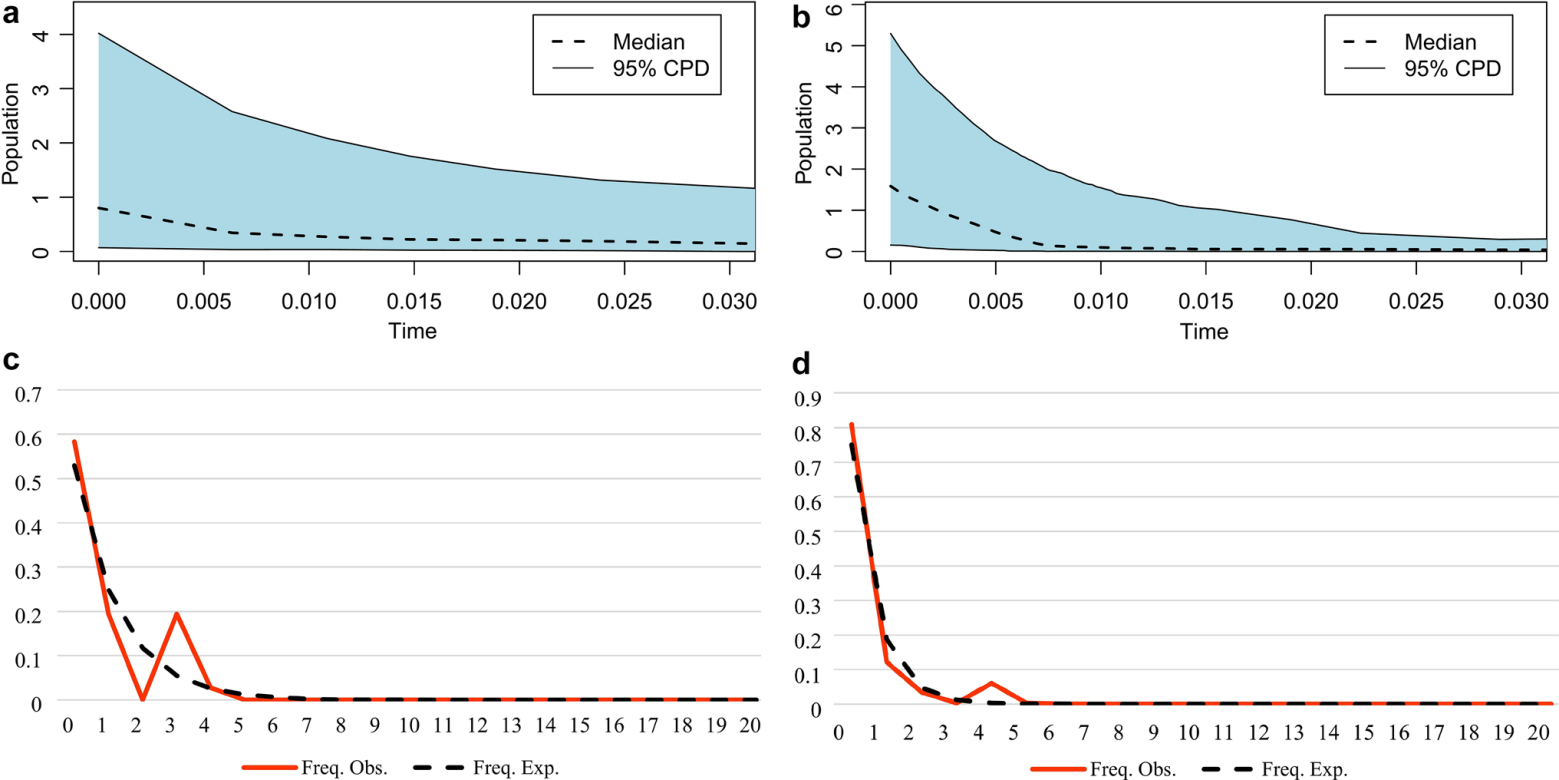
Above: Extended Bayesian skyline plot indicating demographic size changes in the mitochondrial genetic clusters B (**a**) and F (**b**) of the common pheasant from the contact zone of Central and Southeast Europe. Time is given in units of millions of years before present. The central dotted line represents the median value of effective population size, and solid lines indicate the 95% highest posterior density (HPD) interval. Below: Mismatch distributions of the genetic clusters B (**c**) and F (**d**). The solid red curves represent the frequencies of the observed pairwise differences and dotted black lines indicate the expected distributions under a sudden expansion model.

The EBSP indicated an increase in maternal effective population sizes for both detected clusters (B and F) since approximately the last 10,000 years (Fig. 4). The mismatch distribution analysis indicated a unimodal distribution for both clusters B and F (Fig. 4), fitting to the sudden expansion model. The sums of squared differences (SSD) and raggedness indices (H_ri_) were not significant (*P* > 0.05) in any of the cases (Table 1), also supporting population expansion for the population. The values of the Tajima’s D and Fu’s FS statistics were mostly negative for the clusters B and F and total sequences in the study area (Table 1), indicating, respectively, an excess of rare nucleotide variants and of rare haplotypes relative to those expected under mutation-drift equilibrium. While both tests were significant for clade F, only the Tajima's test was significant for clade B.

### Microsatellite analysis

#### Hardy–Weinberg and linkage disequilibrium tests

Of the 10 loci, we did not include the results for two of them in the data set for analysis because one (PC5) was monomorphic and the other (PC1) had a high proportion of missing data. The global test indicated significant deviations from HWE after Bonferroni correction (P values < 0.0009) and heterozygote deficit in three loci (PC4, PC7, and PC10). However, in separate tests for each of the two countries, these three loci showed significant deviation from HWE and heterozygote deficit only for Serbia, not for Hungary. This suggests that the data for these loci may not be affected by intrinsic problems, such as null alleles or allelic dropout, but that the significant test results in Serbia may be due to underlying genetic structure (Wahlund effect). Accordingly, estimates of the frequency of null alleles at these loci were generally small (< 0.2; Table S2). We therefore retained these loci in the data set. No signs of significant linkage disequilibrium between loci were detected after Bonferroni correction, both in the global test and in the analyses separately by country.

#### Genetic structure and population differentiation

**Figure 5:**
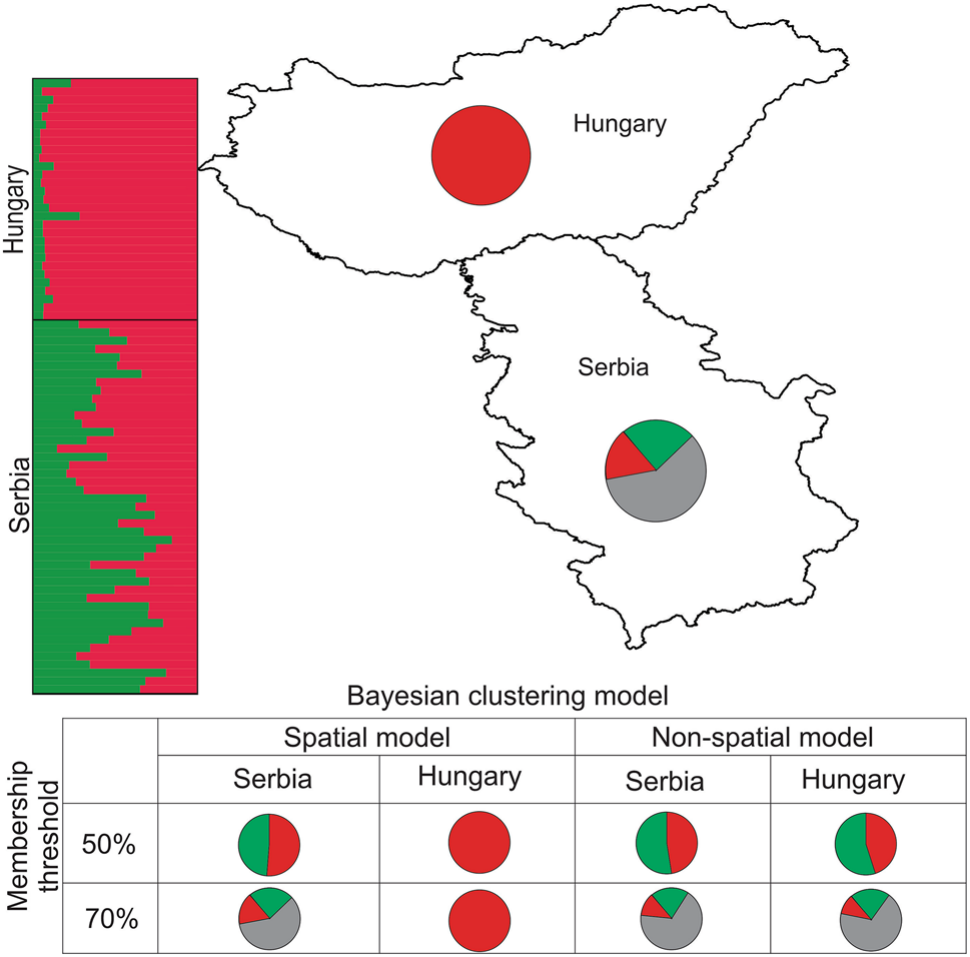
STRUCTURE assignment of common pheasant individuals into two (K = 2) genetic clusters. The bar plot on the left and the pie chart in each country refer to the analyses using information on sampling location and the membership probability threshold of 70%. In the bar plots, each individual is depicted by a column that is partitioned into K segments, which length is proportional to the membership coefficient of the individual for each cluster. Grey in the pie charts represents the percent of individuals that could not be assigned to any cluster. The results of non-spatial model and the membership probability threshold of 50% is given in the attached table. Map source: ESRI. The map was generated using ArcGIS 10.4.1 by ESRI (available online at: http://www.esri.com/).

In the STRUCTURE analysis, both LnP(D) and ΔK suggested the presence of two genetic clusters (K = 2) with either the model with prior information on the sampling location of individuals or the non-spatial model (Fig. 5). With the model using information on sampling location and applying a membership probability threshold of 50%, all Hungarian samples grouped in the same cluster, whereas in Serbia the individuals were approximately divided equally into each cluster (53% in the same cluster as the Hungarians, and 47% in the other). When applying a stricter threshold of 70%, the results for the Hungarian samples were the same as for the 50% threshold, while most Serbian samples (64%) could not be assigned to any cluster (of the remaining 36%, 18% were assigned to one cluster and 18% to the other). The analyses using the non-spatial model did not detect any clear geographic structure in the data. Specifically, considering the 50% threshold for cluster membership, 55% of the Hungarian samples and 53% of the Serbian samples were assigned to the same cluster, with the remaining 45% and 47%, respectively, grouping into the other cluster. With a threshold of 70%, 73% of the Hungarian samples and 76% of the Serbian samples had intermediate assignment probabilities to both clusters. As a great number of Serbian samples were not assigned to any of the two clusters in both spatial and non-spatial models, we studied cluster membership probabilities for models with more than two clusters to understand admixture patterns and fine-scale genetic structuring. However, the spatial models with K > 2 (K = 3, 4, and 5) and membership threshold of 70% showed that none of the admixture Serbian samples identified with K = 2 were assigned to a specific cluster, confirming the absence of discrete subpopulations whthin Serbian samples and high degree of admixture (Figure S2).

**Figure 6:**
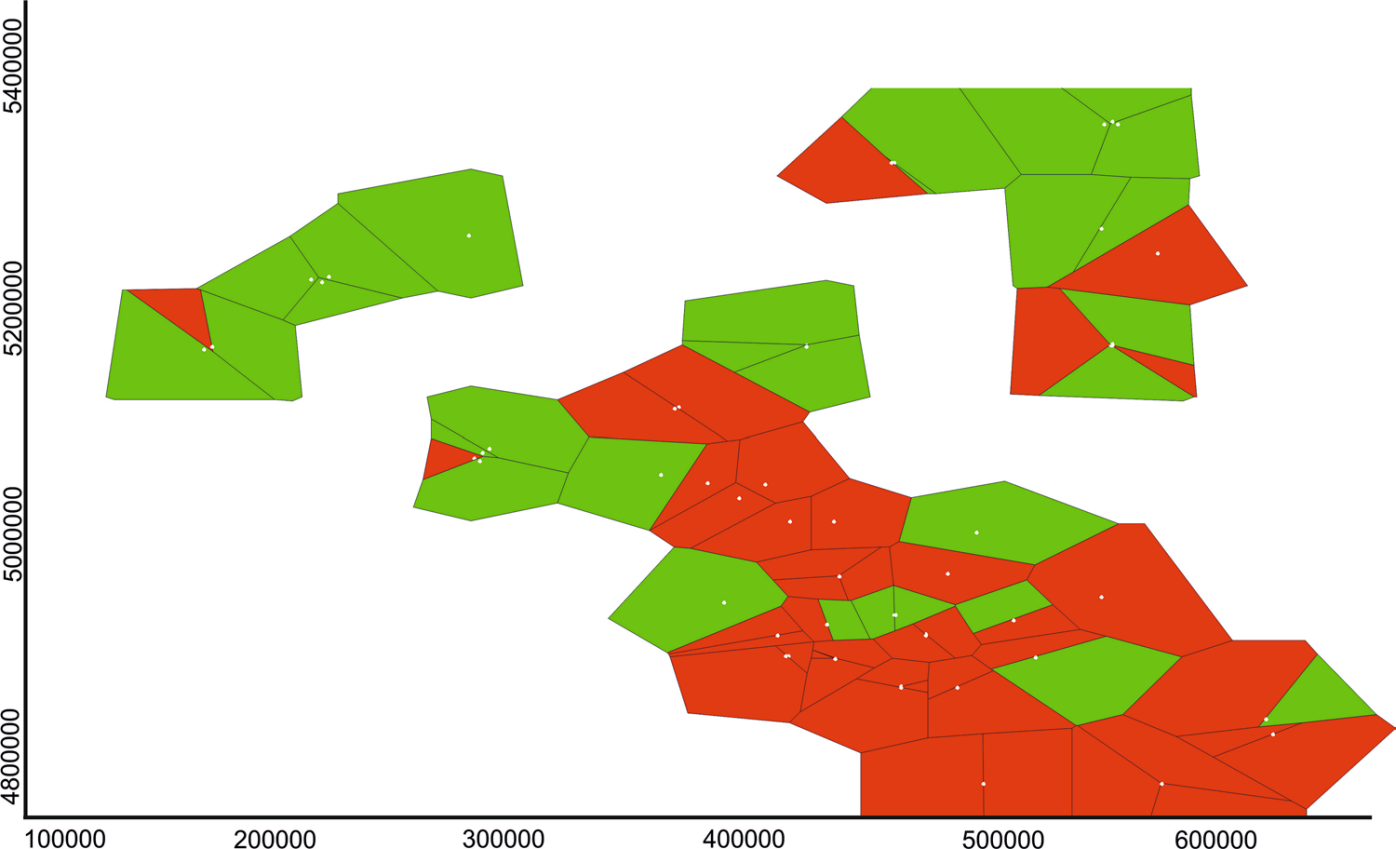
Bayesian spatial clustering of individuals in BAPS for K = 2. The figure shows a Voronoi tessellation of the distribution of sample locations in the study area), in which the tessellation cells are coloured according to cluster membership of samples.

**Figure 7:**
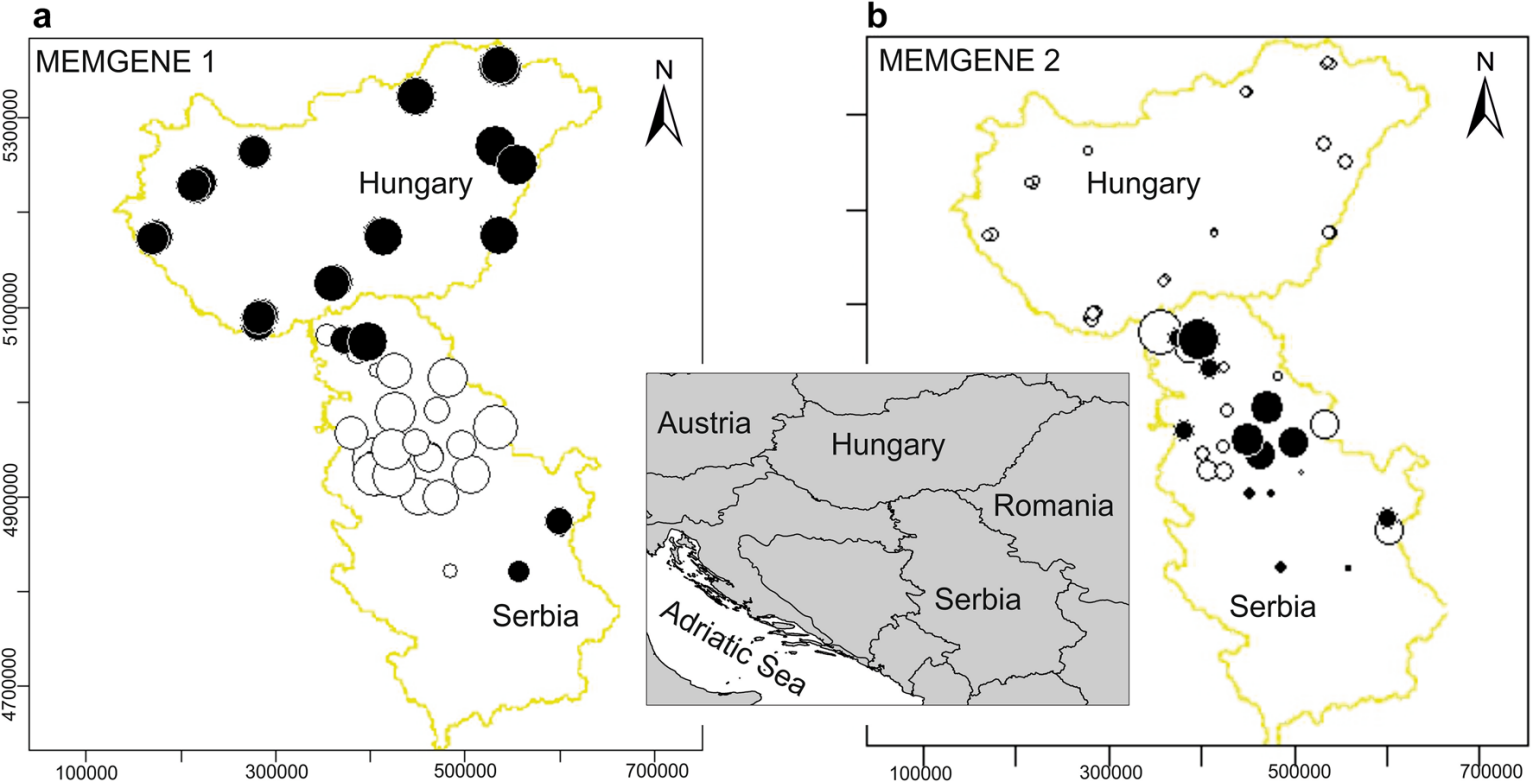
The results of the first two MEMGENE variables (**a** MEMGENE1, **b** MEMGENE2) for 74 common pheasants in Hungary and Serbia. Circles of similar size and colour indicate individuals with similar scores (large black and white circles describe opposite extremes on the MEMGENE axes). Map source: ESRI. The map was generated using ArcGIS 10.4.1 by ESRI (available online at: http://www.esri.com/).

The Bayesian spatial clustering of individuals performed in BAPS also supported the presence of two clusters (K = 2) as the most likely (Fig. 6). Most of the samples from Hungary were allocated to one cluster (shown in green) while the majority of samples from Serbia grouped in the other cluster (in red). The MEMGENE analysis identified two significant variables (Fig. 7). The first (MEMGENE1) suggests a significant genetic structure that mostly corresponds to a differentiation between the common pheasant populations in Hungary and Serbia (Fig. 7a). The second (MEMGENE2) supports the pattern of genetic differentiation between Hungary and Serbia suggested by MEMGENE1, and indicates the presence of genetic mixture in Serbia, which, in turn, appears to be essentially absent in Hungary (Fig. 7b). The estimated amount of genetic structure explained by spatial patterns was modest (R^2^adj = 0.072), indicating that much of the spatial genetic pattern can be explained by IBD.

**Figure 8:**
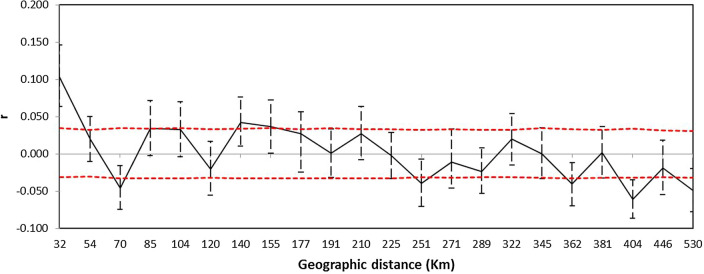
Correlogram of the autocorrelation coefficient r. r is a measure of the genetic similarity between pairs of individuals falling within a given distance class. The red dashed lines represent the 95% confidence envelope for the hypothesis of no spatial genetic structure as determined by permutation. The error bars show the 95% confidence intervals about r obtained by bootstrapping.

The spatial autocorrelation analysis showed significant coefficients (p < 0.01) for the smallest distance class (i.e. 0–50 km; Fig. 8), suggesting an IBD pattern at this spatial scale. A marginally significant positive correlation was also found in the distance class 140–155 km, and no other significant positive correlation was detected in the remaining distance classes. The Mantel test indicated the presence of a modest global pattern of IBD (r = 0.136; p = 0.0004; upper limit of a 95% confidence interval = 0.219. Contraty to the global pattern of IBD in the whole dataset, we didn’t find a significant relation between geographic and genetic distance in the Serbian samples (r = 0.078; p = 0.081).

#### Genetic diversity and bottleneck tests

Across all individuals, a total of 68 alleles were detected, with the number of alleles per locus ranging from four to 19 (mean 8.5), and the mean number of effective alleles, HO and HE were 3.82, 0.580 and 0.651, respectively (Table 3). Estimates of the different genetic diversity parameters were generally similar between the two countries. The most notable differences were in the number of private alleles, which were almost double in Serbia (although it also should be noted that more Serbian samples were analyzed), whereas heterozygosity was higher in Hungary (Table 3).

**Table 3:** Summary statistics of genetic diversity for all samples and by country.

Samples	N	MNA	Ap	AR	NE	HO	HE	PIC	FIS
All	74	8.5	–	8.44	3.82	0.580	0.651	0.410	0.110
Serbia	45	7.62	13	6.93	3.63	0.503	0.609	0.574	0.175
Hungary	29	6.87	7	6.83	3.66	0.701	0.686	0.634	− 0.022

*N* number of samples, *MNA* mean number of alleles, *AP* number of private alleles, *AR* allelic richness, *NE* mean number of effective alleles, *HO* heterozygosity observed, *HE* heterozygosity expected, *PIC* polymorphic information content, *FIS* inbreeding coefficient.

Bold numbers indicate significant values after sequential Holm-Bonferroni correction.

No evidence of a recent genetic bottleneck was detected in any of the two data sets analysed (country-level and cluster-level), and under any of the three tested mutation models, based on the Wilcoxon’s signed rank test, except for the cluster 2 (all Hungarian and 53% of Serbian samples)  under the IAM (which was significant at α = 0.05). Based on the sign test, the Serbia data set showed evidence of a recent genetic bottleneck only under the SMM (Table S3). Given that for fewer than 20 loci the Wilcoxon’s test may be the most appropriate and powerful, and that the TPM appears to be the most appropriate model for most microsatellites^52^, overall we interpret these results as indicating absence of a recent bottleneck. Concordantly, the ‘mode-shift’ test in the three data sets always found a normal L-shaped allele frequency distribution.

## Discussion

Although several studies have investigated the genetic diversity, population structure and demographic history of the common pheasant across its native range in Asia^30,31,43–45^, there is a great lack of this kind of information on the populations introduced into Europe. This study provides the first detailed genetic assessment of common pheasants in their introduced European range, particularly in the contact zone of Central and Southeast Europe (Hungary and Serbia), and the results are essential information for the development of effective conservation and management strategies.

### Mitochondrial diversity, phylogeography and demographic history

Mitochondrial diversity of common pheasants in the study area is relatively low (Hd = 0.192–0.417, π = 0.00041–0.00108), particularly when compared to that reported for native populations in Asia (e.g.^37^); Hd = 0.500–1, π = 0.0009–0.0130). The phylogenetic analysis, including both the new control region sequences generated here and those previously published, inferred the same clades already identified in previous studies (e.g.^30,31^) (Fig. 2). The mitochondrial sequences found in the study area belong to two well-supported clades: B (corresponding to several subspecies groups such as mongolicus, torquatus, and pallasi) and F (corresponding to the colchicus subspecies group)^30,31,44^.

Notably, clade F is much more frequent and geographically widespread, while clade B is mainly present in Serbia (Fig. 1), where the geographic overlap between the two clades is much more significant. Boev^29^ proposed that the first common pheasants introduced into Europe were *colchicus* (as early as 500 AD), and only much later (eighteenth century) other subspecies (*mongolicus* and *torquatus*) would have been introduced. Our results, given the much greater frequency and distribution of clade F in the study area, are consistent with the hypothesis that colchicus was long introduced, so that this lineage had ample time and opportunity to spread throughout the region. Haplotype H1 of clade F and haplotype H2 of clade B were the most frequent and widespread in the study area (Fig. 1). This, and the relatively low number of haplotypes found in the study area, when compared for example to the haplotype richness in clade B (Figs. 2 and 3), suggest introductions involving a small number of individuals, followed by demographic and range expansion. The apparently more recent introduction of clade B seems to have so far spread mainly only across Serbia.

The EBSP results indicated that *P. colchicus* likely experienced a long-term historical expansion based on detected clusters in the study area (Fig. 4). Morever, mismatch distribution analysis suggested evidence for unimodal distribution of the clusters B and F, which is relevant to the panmictic population undergone the sudden expansion model in its evolutionary history^53,54^. In addition, the results of the Fu’s and Tajima’s mutation-drift equilibrium tests and the star-shaped pattern in the haplotype network support the hypothesis of demographic expansion of clade F (Table 1; Fig. 3). Given the postulated timings of the introductions of the common pheasant in Europe^29^ and the time scale of the mtDNA mutation rate, this expansion signal likely reflects an older process that took place in the native range, but it is possible that some of the less frequent haplotypes differing by a single mutation from H1 are derived from mutations that occurred after introduction. Our result supporting the hypothesis of historical demographic expansion in the colchicus group is in line with results in Kayvanfar et al.^31^ and Liu et al.^45^. Also, we find evidence for the hypothesis of historical demographic expansion for clade B. This hypothesis is suggested for the torquatus group by several previous studies^30,31,42,45^.

### Microsatellite diversity, genetic structure and bottleneck tests

The estimated moderate levels of microsatellite genetic diversity for common pheasants in the study area were similar to those reported for a sample of comparable size containing equal numbers of nine subspecies from China^55^. Therefore, possible genetic signals in the microsatellite diversity of founder effects associated with introductions (indicated by the relatively low nucleotide and haplotype diversity, Table 1^56^) may have been erased as a result of a combination of factors such as the high mutation rate of microsatellites, rapid population growth, and the genetic enrichment resulting from the mixture of subspecies in the study area (e.g. high number of private alleles in Serbia, Table 3). Accordingly, the patterns of microsatellite variability did not show signs of effects of genetic bottlenecks.

Analyses of the microsatellite data using spatial Bayesian clustering and spatial genetic neighbourhood methods consistently suggested a differentiation between Hungary and Serbia, with the common pheasant population in Hungary being much more genetically homogeneous, while that of Serbia has much more genetic mixture and admixture (Figs. 5, 6, and 7). This cryptic differentiation was not detected using a non-spatial Bayesian clustering model. Therefore, the genetic differentiation between the common pheasant populations of the two countries is shallow. This may be due to gene flow resulting from the mobility of the species, since birds, due to their dispersal abilities, often show less population genetic structure than other vertebrates^57–60^. A similar pattern of low genetic structure was observed among populations of common pheasant in China^55^ and of silver pheasant in southern China^61^. Given that the European range of *P. colchicus* is larger than the contact zone, it may be worth noting at some point that studying the species at the European range scale may provide deeper insights into the population structure of the species. We found evidence of some influence of isolation-by-distance on the genetic structure. In particular, spatial autocorrelation analyses indicated non-random distribution of genotypes at scales up to 50 km. Further study is needed to investigate the issue of fine-scale genetic structure.

### Conservation management implications

An important conclusion from our data, both mitochondrial and nuclear, is the fact that they strongly indicate that releases over time to the present day of captive-bred individuals have apparently not led to the establishment of other lineages/subspecies distinct from clades B and F. Also, the detection of neighborhood size up to a distance of 50 km is of practical relevance for the species’ management. Thus, wildlife management practice has traditionally treated this distance value as the basis for isolating pheasant populations. However, the known home range of common pheasants is much smaller: on average 0.05–0.1 km^2^ (range of 0.008–0.215 km^2^), and even less in the breeding season (0.02 km^2^)^62,63^. Our results, however, suggest that the scale of the genetic impact of releases of captive-bred individuals may be much larger than could be predicted by game managers based on the typical home range of the species. It has been observed and reported that the common pheasant populations of the two countries in the study area have been declining for the past 30 years. It is then apparent that the intended population reinforcements with captive-bred individuals have not helped to achieve the impact hoped for by wildlife farmers and could not prevent the decline in population size. However, in the absence of reference areas and populations, it is difficult to judge the exact impact of artificial releases. It may have even helped to strengthen natural populations, but at the same time it could only slowed the rate of population decline. But it may also have been negatively affected the population size trend by outbreeding depression, the introduction and faster spread of diseases and parasites due to birds introduced from foreign sources and the larger herd size, the increase in the number of predators and the neglected natural population due to supplementation.The results of this study provide essential information for conservation management, monitoring, and for comparison with future assessments of the genetic status of common pheasant populations in Central and Southeast Europe.

## Material and methods

### Sample collection and DNA extraction

We collected 74 fresh tissue samples in the contact zone of Central and Southeast Europe (Hungary and Serbia) between 2014 and 2015 (Fig. 1). Figures S3 and S4 show, respectively, wild-born and captive-bred pheasants from Hungary. The samples were obtained from legally hunted pheasants and stored in 96% ethanol. No animals were shot only for the purpose of this study. An ethics statement was not recquired for this work; collection was done in accordance with the countries’ national regulations and under hunting license. Genomic DNA was extracted using Roche High Pure PCR Template Preparation Kit (Roche Life Sciences) following the manufacturer's protocol.

### Mitochondrial DNA control region sequencing

A 825-bp fragment of the mitochondrial control region in 69 common pheasants was amplified using the primers PHDL (5’-AGGACTACGGCTTGAAAAGC-3’) and PHDH (5’-CATCTTGGCATCTTCAGTGCC-3’)^64^. Polymerase chain reactions (PCR) were carried out in a total volume of 25 μL, with the reaction mixture containing 30 to 50 ng of genomic DNA, 10 pmol/L of each primer, 1X PCR buffer (Qiagen, USA), 6.25 mmol/L MgCl2, 2.5 units of Taq polymerase (Qiagen, USA), and 10 pmol/L dNTPs. The PCR conditions were as follows: an initial denaturation at 94 °C for 2 min, followed by 30 cycles of 15 s at 94 °C, 15 s at 55 °C and 1 min at 72 °C, and a final extension for 10 min at 72 °C. PCR products were run on 2% agarose gels, stained with ethidium bromide, and visualized under ultraviolet (UV) light. PCR product purification and sequencing were carried out by Macrogen Europe. Sequences were assembled using SeqScape v.2.6 (Applied Biosystems) and aligned with ClustalW in MEGA 6.0^65^. The substitution saturation was examined in DAMBE 6^66^.

### Microsatellite genotyping

Ten microsatellite loci (Table S4), previously developed for *P. colchicus* by Wang et al.^55^, were used to genotype 74 individuals from the study area. The PCRs consisted of 12.5 μL of Multiplex PCR Master Mix (QIAGEN), 0.3 μL of each primer (10 pmol/μL), 1 μL genomic DNA (15 ng/μL), and sterile water up to a final volume of 25 μL. Thermal cycling consisted of a 5 min initial denaturation at 95 °C, followed by 35 cycles consisting of denaturation at 94 °C for 30 s, annealing at 58 °C for 45 s, and extension at 72 °C for 1.5 min, and then a final extension at 72 °C for 10 min. The forward primers were labelled at the 5′ end with either 6-FAM, VIC, NED or PET fluorescent dyes (Table S4). Fragment analysis was performed on an ABI 3730XL DNA Analyser (Applied Biosystems). Genotypes were determined using Peak Scanner v1.0 (Applied Biosystems).

### Mitochondrial DNA analysis

#### Genetic diversity and phylogenetic analyses

New sequences in the present study were combined with 202 GenBank sequences from previous studies (Table S1) to yield a dataset in order to estimate the phylogenetic relationships among common pheasants across the species range. The number of haplotypes, haplotype diversity (Hd), nucleotide diversity (π), and the number of polymorphic sites (P) were calculated using DnaSP 5.10.1^67^. The best-fit models of nucleotide substitution were selected using jModeltest 0.1.1^68^ under the corrected Akaike Information Criterion (AICc) and the Bayesian Information Criterion (BIC). IQ-TREE 1.6.12^69^ was used to infer a maximum likelihood (ML) tree under a HKY + I + G model^70^, and clade support was assessed using 10,000 bootstrap replicates. BEAST 2.6.4^71^ was used to reconstruct a Bayesian inference (BI) tree under a HKY + I + G model, based on 40 million generations of Markov Chain Monte Carlo (MCMC) chains and sampling every 100 generations. Three sequences of the green pheasant (*Phasianus versicolor*) (GenBank accession numbers AY376861, AY376862 and AY376863) (Zhan and Zhang, unpublished) were included as outgroups. A median-joining network^72^, estimated in Network 10.0 (available at: http://www.fluxus-engineering.com), was used to infer and visualize possible genealogical relationships among haplotypes.

#### Population structure

We used the Bayesian model-based clustering algorithm implemented in BAPS 6.0 (Bayesian Analysis of Population Structure)^73^, to estimate the presence of mitochondrial genetic clusters within the new sequences of *P. colchicus*. BAPS was run setting the method of ”clustering for linked loci” with two independent runs, and the maximum number of clusters (K) to 10. To examine genetic differentiation among clusters, analysis of molecular variance (AMOVA) and fixation indexes (F_ST_) were calculated using Arlequin 3.5.2.2^74^ with 10,000 permutations to test for significance.

#### Demographic analyses and neutrality tests

We constructed extended Bayesian skyline plots (EBSPs)^75^ in BEAST 2.6.4^71^ to explore the demographic dynamics of detected population of the common pheasant in the study area (clusters B and F detected by BAPS). The latter analyses were conducted using a HKY substitution model, a strict clock model, and the coalescent extended Bayesian skyline as tree prior. The MCMC chain was set to a total 400 million generations, and the Markov chain was sampled every 10,000 steps. Tracer was used to assess the convergence and stationarity of the EBSP values, and whether the effective sample sizes (ESS) were > 200. We performed multiple replicate runs to ensure MCMC convergence. EBSPAnalyzer 2.5.2^71^ was employed to construct the EBSPs. We used R 3.1.2^76^ to generate the visualizations.

Mismatch distribution analyses were used to examine demographic expansions in detected population clusters. To compare observed distributions with expected distributions under the expansion model, we estimated sums of squared deviations (SSD) and Harpending's raggedness index (RI) in Arlequin. Morever, we used the tests of Fu’s FS^77^ and Tajima’s D^78^, computed in Arlequin to test equilibrium of the population. A negative value of these statistics signify an excess of low frequency polymorphisms, and suggests a population size expansion and/or purifying selection of a population^78^. When we detect more than one distinct population cluster in the BAPS analysis, we carried out the EBSP analyses, mismatch distribution and neutrality tests for each cluster separately.

### Microsatellite analysis

#### Hardy–Weinberg and linkage disequilibrium tests

We used exact tests in Genepop 4.2^79^ to test loci for Hardy–Weinberg equilibrium (HWE) deviations and linkage disequilibrium (LD) between pairs of loci, based on the whole dataset and separately for each region (Hungary and Serbia). Probability values were assessed for significance using sequential Bonferroni correction for multiple comparisons and a nominal significance value of 0.05^80^.

#### Genetic structure and population differentiation

We used STRUCTURE 2.3.4^81^ to investigate the likely number of genetic clusters (K) in the study area. STRUCTURE was run using the admixture ancestry model, correlated allele frequencies, K from 1 to 5, 10 independent simulations for each K, 100,000 iterations as burn-in, and 1,000,000 iterations for sampling. We conducted Bayesian clustering with (i.e. LOCPRIOR model; assuming two geographic populations; (Serbia and Hungary)^82^) and without prior information on sampling locations. We used STRUCTURE HARVESTER^83^ to process and visualize results from STRUCTURE, and we determined the most likely value of K based on the log probability of the data (Ln P(D)) and the ΔK statistic^84^. The membership probabilities (Q-values) were used to assign individuals to clusters considering a membership threshold of either 50% or 70% (e.g.^85,86^). CLUMPP 1.1.2^87^ and DISTRUCT 1.1^88^ were used to average the membership probabilities over the 10 runs for each K and to plot the results, respectively. The number of genetic clusters present in the study area and the degree of admixture between them was also investigated using the spatial mixture model in BAPS 6^73,89^. We tested K values from 1 to 5, and subsequently performed a separate analysis in the ‘fixed K’ mode with K = 2. Additionally, we used MEMGENE 1.0.1^90^ to complement the analyses with STRUCTURE and BAPS and to visualize spatial genetic neighbourhoods. MEMGENE performs a spatially explicit ordination technique free of linkage and Hardy-¬Weinberg equilibria assumptions; in particular it uses Moran’s Eigenvector Maps (MEM) to detect weak genetic structure^90^. Spatial autocorrelation analyses were used to detect fine-scale patterns of genetic structure across the study area^8,91,92^. We divided the individuals into 22 geographic distance classes of equal sample size. A correlation coefficient r between geographic and genetic distance was calculated among individuals in each class. Significance tests were performed using 10,000 permutations to test the null hypothesis of no spatial autocorrelation. Confidence intervals around the estimates of r were determined using bootstrapping with 10,000 replicates. The results were plotted in a correlogram. Finally, we used the Mantel test within the R package Vegan version 2.5-7^93^ to assess the hypothesis of isolation-by-distance (IBD)^94^. For this, we calculated genetic and log-transformed geographic distances among all pairs of individuals in GenAlEx 6.5^95^. A total of 10,000 replicates were used to determine the significance of the test relative to the 95% quantile of the distribution of permutations.

#### Genetic diversity and bottleneck tests

We quantified genetic diversity using basic genetic variation statistics, including observed (HO) and expected (HE) heterozygosity, mean number of alleles (MNA), allelic richness (AR), effective number of alleles (NE), private allele frequency (AP), and polymorphic information content (PIC). The estimates and 95% confidence intervals were obtained for the total sample and separately for each country (Hungary and Serbia) using GenAlEx, FSTAT v.2.9.3.2^96^, and POPGENE 1.32^97^. The inbreeding coefficient (FIS) was estimated using GENETIX4.05^98^ and the significance of values was evaluated based on 10,000 permutations. BOTTLENECK v1.2.0269^52^ was used to test for evidence of recent bottlenecks in effective population size at two levels including cluster-level (STRUCTURE-inferred clusters based on the spatial model and membership probability threshold of 50%) and country-level (Hungary and Serbia). We ran BOTTLENECK using 1000 iterations and three different mutation models: the infinite alleles model (IAM), the stepwise mutation model (SMM), and the two-phase model (TPM; variance for TPM = 12% and proportion of SSM in TPM = 95%, following recommendations of Piry et al.^52^ for microsatellites). The statistical tests applied in BOTTLENECK were the Wilcoxon signed-rank test^99^, sign test, and the ‘mode-shift’ indicator^100^.

## Supplementary Information

Supplementary Figure S1.

Supplementary Figure S2.

Supplementary Figure S3.

Supplementary Figure S4.

Supplementary Legends.

Supplementary Table S1.

Supplementary Table S2.

Supplementary Table S3.

Supplementary Table S4.

## Data Availability

All haplotypes are available from the Genbank database (accession number (s) KY474094- KY474103 for the mtDNA control region.
