# Supplementary figures and images for: Assessing the origin, genetic structure and demographic history of the common pheasant (Phasianus colchicus) in the introduced European range
Source: Sci Rep. 2021 Nov 5;11:21721. doi: 10.1038/s41598-021-00567-1 (PMC8571287; doi:10.1038/s41598-021-00567-1)

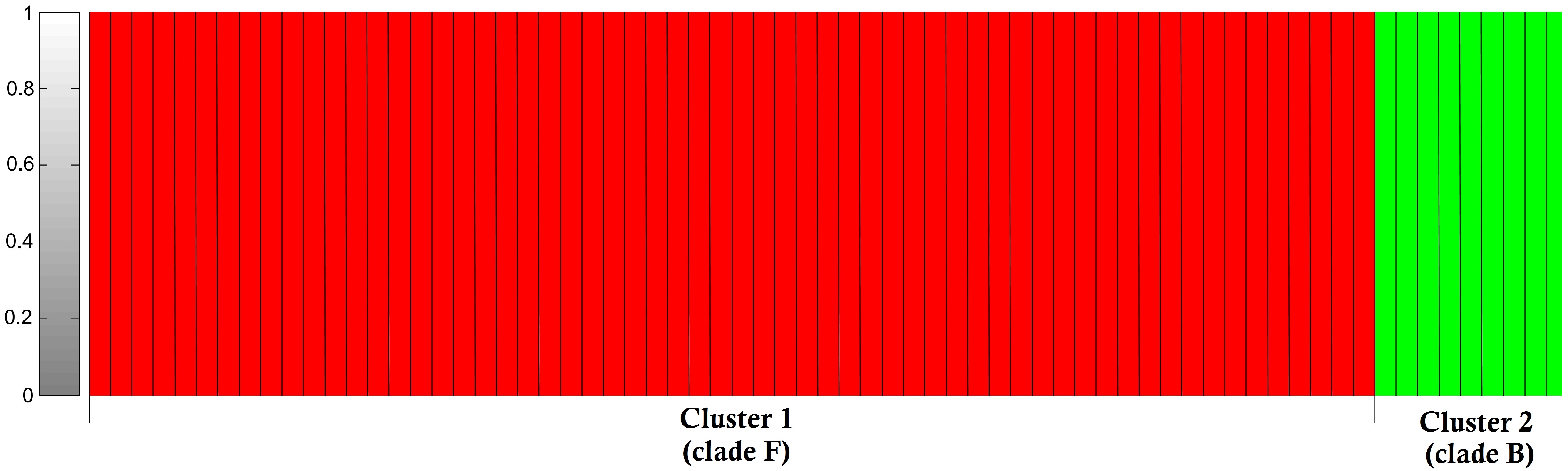

Supplement: Supplementary file 1 — Supplementary Figure S1. [file 41598_2021_567_MOESM1_ESM.jpg]

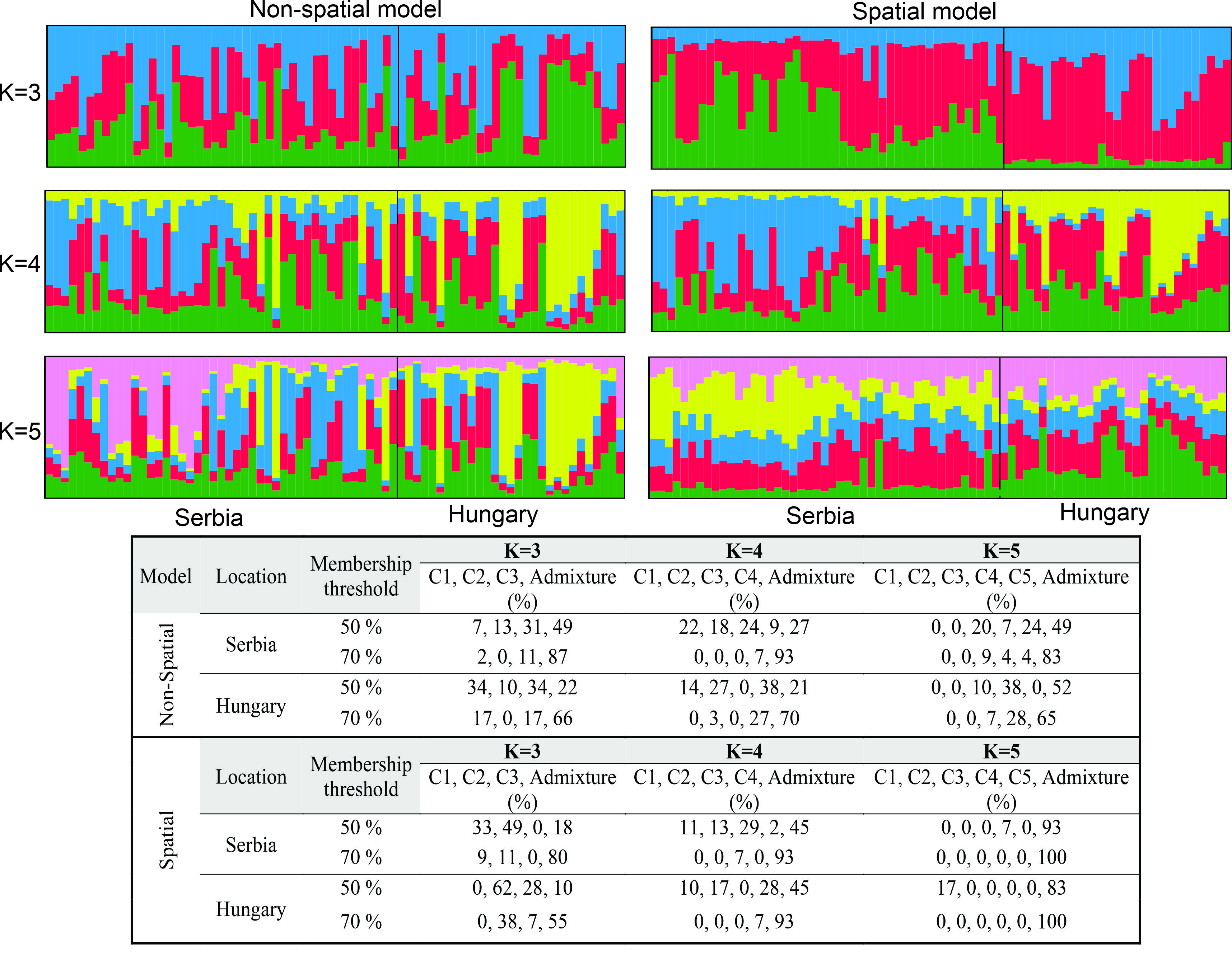

Supplement: Supplementary file 2 — Supplementary Figure S2. [file 41598_2021_567_MOESM2_ESM.jpg]

**
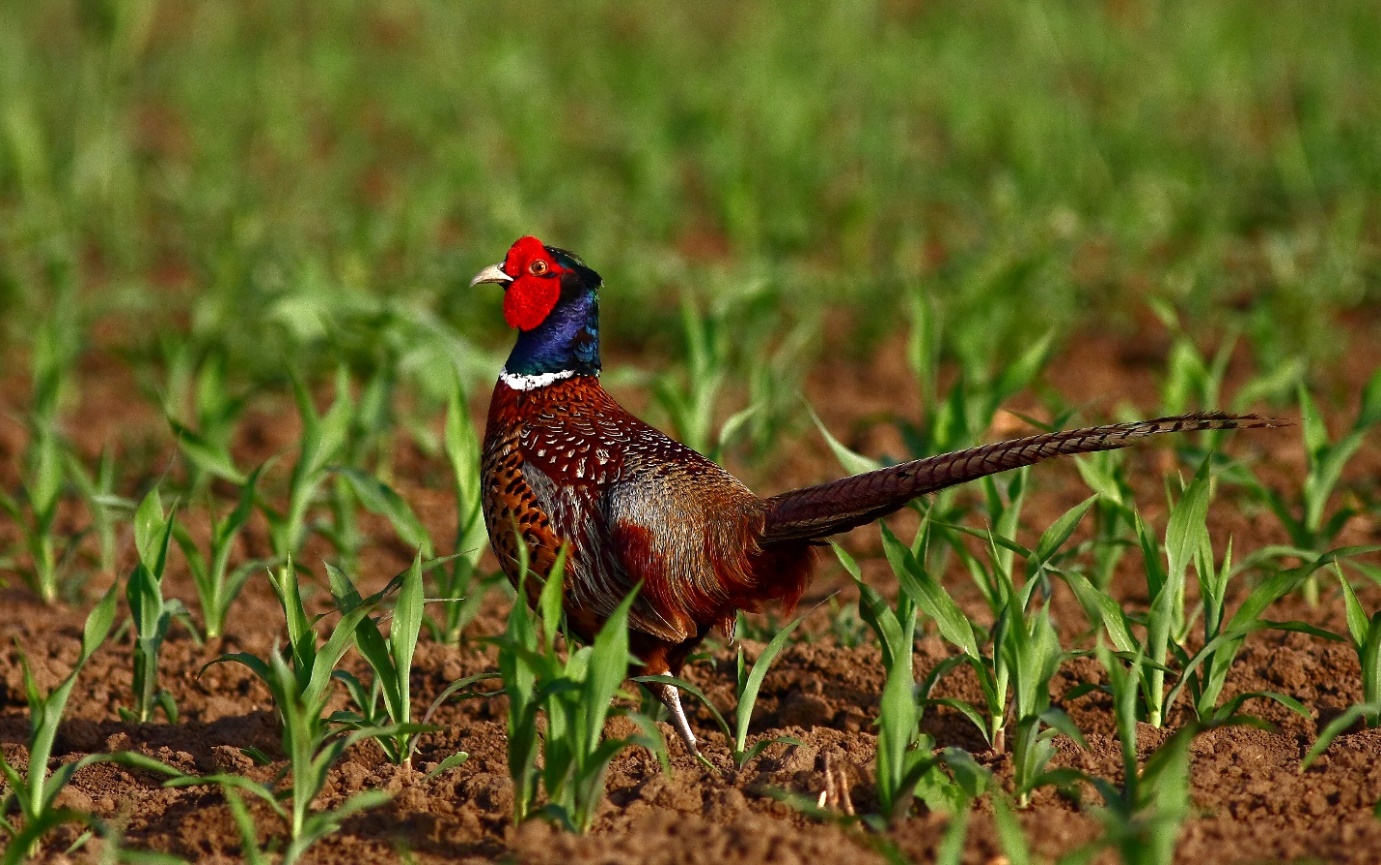
**

Figure S3. Wild-born pheasant from Hungary (Photo: Szendrei, L.)

Supplement: Supplementary file 3 — Supplementary Figure S3. [file 41598_2021_567_MOESM3_ESM.docx]

**
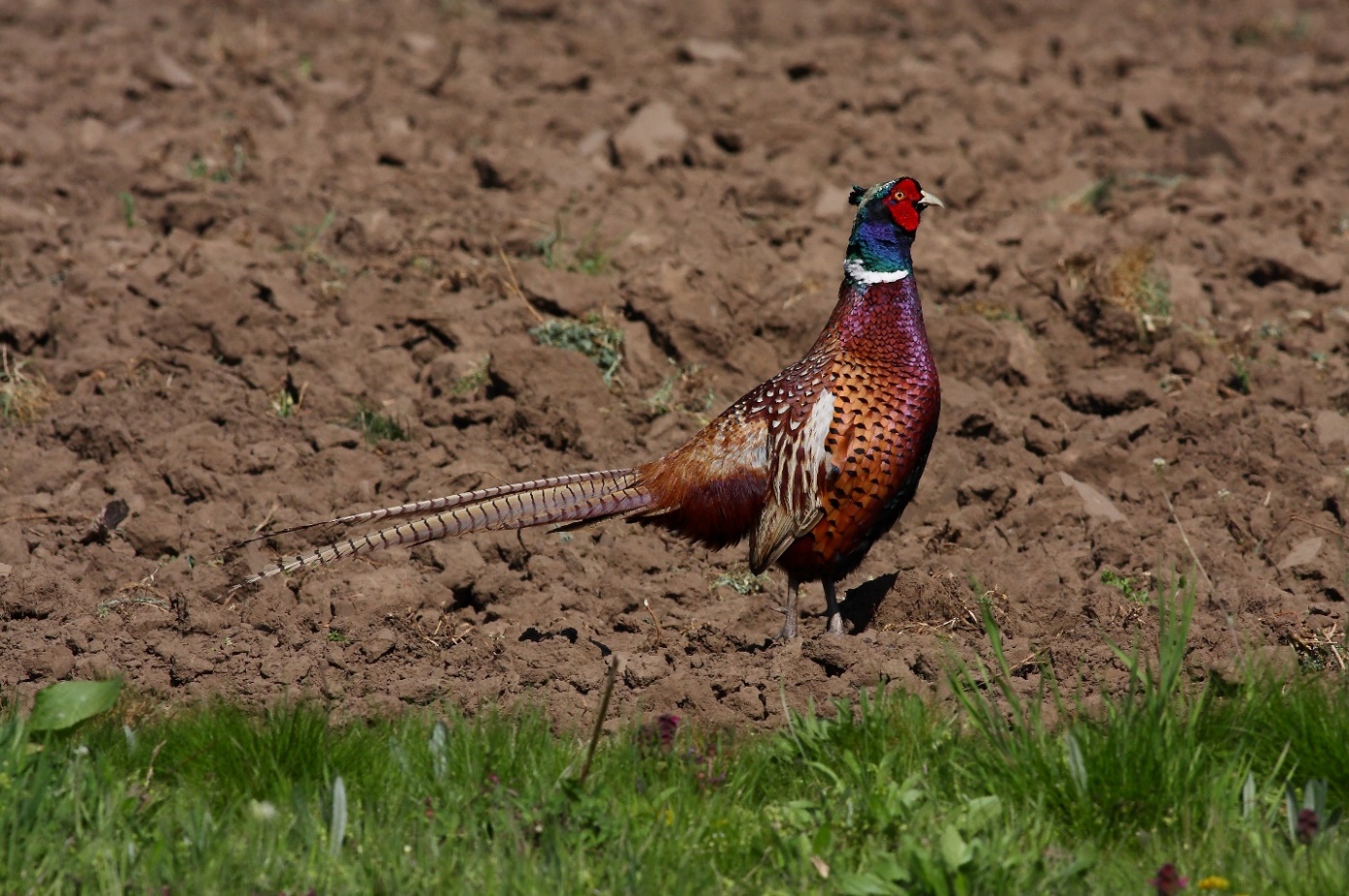
**

Figure S4: Captive-bred pheasant from Hungary (Photo: Szendrei, L.)

Supplement: Supplementary file 4 — Supplementary Figure S4. [file 41598_2021_567_MOESM4_ESM.docx]
