# Supplementary material for: Assessing the origin, genetic structure and demographic history of the common pheasant (Phasianus colchicus) in the introduced European range
Source: Sci Rep. 2021 Nov 5;11:21721. doi: 10.1038/s41598-021-00567-1 (PMC8571287; doi:10.1038/s41598-021-00567-1)
Supplement: Supplementary file 5 — Supplementary Legends. [file 41598_2021_567_MOESM5_ESM.docx]

**Supplementary Information**

**Figure S1:** Bayesian clustering analysis of common pheasant using 825-bp fragment of the mitochondrial control region as implemented in BAPS v6. resulting in K = 2.

**Figure S2**: STRUCTURE assignment of common pheasant individuals into three (K=3), four (K=4), and five (K=5) genetic clusters (C1, C2, C3, C4, C5) based on the membership probability threshold of 50% and 70%. The bar plots on the right and left refer to spatial and non-spatial models, respectivelly. In the bar plots, each individual is depicted by a column that is partitioned into K segments, which length is proportional to the membership coefficient of the individual for each cluster.

**Figure S3**: Wild-born pheasant from Hungary (Photo: Szendrei, L.)

**Figure S4**: Captive-bred pheasant from Hungary (Photo: Szendrei, L.)

**Table S1.** Details of sequences used in the phylogenetic analysis of common pheasant.

**Table S2.** Estimated null allele frequencies for the eight microsatellite loci using FreeNA.

**Table S3.** The results of bottleneck test for recent reduction in populations of the common pheasant (*Phasianus colchicus*) in the introduced European range. IAM: infinite alleles model; TPM 95%: two-phase model with 95% single-step mutations; SMM: stepwise mutation model. Significant p values of heterozygosity deficiency are bolded.

**Table S4.** Information on the 10 microsatellite loci used in this study.
