# Supplementary material for: Assessing the origin, genetic structure and demographic history of the common pheasant (Phasianus colchicus) in the introduced European range
Source: Sci Rep. 2021 Nov 5;11:21721. doi: 10.1038/s41598-021-00567-1 (PMC8571287; doi:10.1038/s41598-021-00567-1)
Supplement: Supplementary file 7 — Supplementary Table S2. [file 41598_2021_567_MOESM7_ESM.docx]

**Table S2.** Estimated null allele frequencies for the eight microsatellite loci using FreeNA.

| **Whole samples** | **Hungary** | **Serbia** | **Locus** |
| --- | --- | --- | --- |
| 0.139 | 0.112 | 0.137 | PC4 |
| 0.017 | 0.001 | 0.009 | PC6 |
| 0.172 | 0.145 | 0.169 | PC7 |
| 0.000 | 0.000 | 0.003 | PC3 |
| 0.050 | 0.056 | 0.032 | PC8 |
| 0.052 | 0.000 | 0.092 | PC10 |
| 0.094 | 0.126 | 0.025 | PC2 |
| 0.000 | 0.000 | 0.037 | PC9 |
