# Supplementary material for: Assessing the origin, genetic structure and demographic history of the common pheasant (Phasianus colchicus) in the introduced European range
Source: Sci Rep. 2021 Nov 5;11:21721. doi: 10.1038/s41598-021-00567-1 (PMC8571287; doi:10.1038/s41598-021-00567-1)
Supplement: Supplementary file 8 — Supplementary Table S3. [file 41598_2021_567_MOESM8_ESM.docx]

**Table S3.** The results of bottleneck test for recent reduction in populations of the common pheasant (*Phasianus colchicus*) in the introduced European range. IAM: infinite alleles model; TPM 95%: two-phase model with 95% single-step mutations; SMM: stepwise mutation model. Significant *p* values of heterozygosity deficiency are bolded. The cluster-level data set is based on the spatial model and membership probability threshold of 50% in STRUCTURE.

|  | **Area** | **Mutation model** | **Sign test** | **Wilcoxson test** |
| --- | --- | --- | --- | --- |
| **Country-level** | **Serbia** | I.A.M. | 0.082 | 0.097 |
|  |  | T.P.M. 95% P | 0.066 | 0.994 |
|  |  | S.M.M. | **0.000** | 1.000 |
|  | **Hungary** | I.A.M. | 0.293 | 0.098 |
|  |  | T.P.M. 95% P | 0.424 | 0.726 |
|  |  | S.M.M. | 0.055 | 0.973 |
| **Cluster-level** | **Cluster 1**  (47% of Serbian samples) | I.A.M. | 0.533 | 0.679 |
|  |  | T.P.M. 95% P | 0.073 | 0.994 |
|  |  | S.M.M. | 0.079 | 0.994 |
|  | **Cluster 2**  (All Hungurian and 53% of Serbian samples) | I.A.M. | 0.299 | **0.019** |
|  |  | T.P.M. 95% P | 0.189 | 0.902 |
|  |  | S.M.M. | 0.188 | 0.980 |
