# Supplementary material for: Assessing the origin, genetic structure and demographic history of the common pheasant (Phasianus colchicus) in the introduced European range
Source: Sci Rep. 2021 Nov 5;11:21721. doi: 10.1038/s41598-021-00567-1 (PMC8571287; doi:10.1038/s41598-021-00567-1)
Supplement: Supplementary file 9 — Supplementary Table S4. [file 41598_2021_567_MOESM9_ESM.docx]

**Table S4**. Information on the 10 microsatellite loci used in this study.

| **Locus** | **Forward and Reverse Sequence (5′-3′)** | **Fluorescent dye** | **Annealing Temperature (°C)** | **Fragment range (bp)** | **Multiplex for Fragment Analysis** |
| --- | --- | --- | --- | --- | --- |
| PC01 | F:AGCACATCACAGTGCTTTGAGC  R:TTTGCTCAGGAAAAGAAAATAAAGACA | NED | 58 | 201–205 | 1 |
| PC02 | F:AAAAAGCTCATTTGCTGTGGAA  R:TCTTTGTCTTCACCCTCATGGA | PET | 56 | 227–240 | 1 |
| PC03 | F:GAGGGTAGAGAGAAACAGGTGTTGA  R:GAGGTAATCTCTCACTGCTGATTGG | 6-FAM | 57 | 152–167 | 1 |
| PC04 | F:TTCCAAAAGCATATCCCAGAGC  R:TAAGATAGCCCATCCTTTGGGG | VIC | 58 | 87–94 | 1 |
| PC05 | F:TGACCACTACAGTTTCCCATTCTTC  R:AGATCTTCAGTAGCTCTTGGAACACA | PET | 57 | 284–286 | 1 |
| PC06 | F:ACGGTCAGTAAGCATGTACCCC  R:AGCAGTCAATGGAGAGCAGGTT | PET | 57 | 84-101 | 1 |
| PC07 | F:GGCTGTCCTTTTAGCTACAGCAG  R:CATCATCAAGAAGCATTGCAAAA | 6-FAM | 57 | 89-93 | 1 |
| PC08 | F:GACCTCTGTCATTGGTTTTGGA  R:TATGATTGTGAACAGCTGCCAA | PET | 56 | 180-202 | 1 |
| PC09 | F:AATGGGAACTTTTTCAGGGACAA  R:TTTGAAGTTGGTGGGACTCCAT | NED | 58 | 237-270 | 1 |
| PC10 | F:GCTGCAAATCTCCTTAGCTCCA  R:GGAGCAACAGTGGGAGAAGAAA | VIC | 58 | 207-242 | 1 |
